# Central and peripheral dimensions of fibromyalgia: a multivariate clinical profiling of migraine and small fibre pathology

**DOI:** 10.1097/PR9.0000000000001510

**Published:** 2026-10-28

**Authors:** Marina de Tommaso, Elena Ammendola, Livio Clemente, Giulia Paparella, Giuseppe Libro, Giulia Paparella

**Affiliations:** a Neurophysiopathology Unit, Pain and Headache Centre, Department of Basic Medical Sciences, Neuroscience and Sense Organs, Bari Policlinico General Hospital, University of Bari Aldo Moro, Bari, Italy; b IRCCS Neuromed, Pozzilli, Italy

**Keywords:** Fibromyalgia, Chronic migraine, Small fibre pathology, Central sensitization, Intraepidermal nerve fibre density, Clinical phenotype

## Abstract

**Introduction:**

Fibromyalgia is a heterogeneous chronic pain disorder involving both central and peripheral mechanisms. Migraine is considered a disorder with abnormal central multimodal and pain stimuli processing, whereas small fibre pathology (SFP) reflects peripheral nociceptive dysfunction. We investigated whether migraine and SFP identify distinct clinical profiles within the fibromyalgia spectrum.

**Methods:**

In this retrospective cross-sectional study, 296 patients with fibromyalgia underwent standardized clinical assessment and skin biopsy for intraepidermal nerve fibre density (IENFD). Migraine was classified as low-frequency episodic, high-frequency episodic, or chronic. Clinical severity was assessed in pain, disability, associated symptoms, and psychological domains. Multivariate analysis of covariance evaluated the independent and combined effects of migraine subtype and SFP. Subgroup analyses and correlation of clinical symptoms with IENFD were performed.

**Results:**

Migraine was diagnosed in 138 patients (46.6%; 69 low-frequency episodic, 28 high-frequency episodic, and 41 chronic migraine). Reduced IENFD was detected in 191 patients (64.5%), and its prevalence did not differ across migraine subtypes. The primary multivariate analysis of covariance did not demonstrate significant multivariate effects of migraine subtype, small fibre pathology, or their interaction. Exploratory subgroup analyses identified higher disability and associated symptom scores in patients with concomitant chronic migraine and small fibre pathology.

**Conclusions:**

Chronic migraine is associated with greater clinical burden in fibromyalgia, whereas small fibre pathology does not identify a distinct multidimensional clinical phenotype. Although patients with concomitant chronic migraine and small fibre pathology showed greater disability and associated symptom burden in exploratory analyses, these findings require confirmation in prospective studies.

## Abstract 2

Supplemental Digital Content is Available in the Text.

In 296 patients with fibromyalgia, chronic migraine identified the subgroup with the greatest clinical burden, whereas concomitant small fibre pathology was associated with an even greater burden of disability and associated symptoms.

## 1. Introduction

Fibromyalgia (FM) is a chronic pain disorder characterised by widespread musculoskeletal pain, fatigue, sleep disturbances, cognitive symptoms, and a broad spectrum of somatic complaints, resulting in substantial disability and impaired quality of life.^25^ Although traditionally considered a disorder of central pain processing, growing evidence indicates that FM is biologically heterogeneous, with variable contributions of central sensitization, peripheral small fibre abnormalities, and psychological factors.^8,17,23^

Migraine is one of the most frequent neurological comorbidities of FM, with a lifetime prevalence approaching 50% to 60%, whereas chronic migraine is present in approximately 10% of patients.^4,14,19^ Conversely, patients with migraine and FM comorbidity have an increased risk of migraine chronification, greater disability, and poorer response to preventive treatments.^7,10^ Because chronic migraine is characterised by persistent central sensitization and enhanced pain facilitation,^6^ its coexistence with FM may identify patients with a greater burden of central nervous system dysfunction.

Another important source of heterogeneity in FM is small fibre pathology (SFP). Reduced intraepidermal nerve fibre density (IENFD) has been reported in approximately half of the patients with FM, suggesting that peripheral nociceptive abnormalities contribute to pain generation in a substantial subgroup.^8,21,23^ Rather than representing a classical peripheral neuropathy, SFP in FM is thought to provide abnormal afferent input capable of maintaining or amplifying central sensitization.^23^ Likewise, although migraine has traditionally been regarded as a disorder of altered central pain processing, activation of the trigeminovascular system and calcitonin gene-related peptide (CGRP)–mediated mechanisms demonstrate that peripheral and central mechanisms interact throughout the disease course.^13,16^

Despite increasing recognition of both migraine and SFP in FM, their relationship remains poorly understood. It is unknown whether these conditions identify distinct clinical phenotypes or whether they represent complementary biological mechanisms contributing to disease heterogeneity. Clarifying this issue may have important clinical implications, as effective prevention of migraine chronification and the availability of mechanism-based therapies, including CGRP-targeted treatments, could modify the overall clinical burden of selected FM subgroups.^9,12,22^

We therefore hypothesized that migraine comorbidity and small fibre pathology represent complementary dimensions of FM heterogeneity rather than mutually exclusive phenotypes. Specifically, we postulated that migraine, according to its frequency and severity, would identify the subgroup with the greatest clinical burden and that concomitant SFP would further amplify disability and associated symptoms.

Accordingly, the aims of this study were to (1) determine the frequency of episodic, high-frequency and chronic migraine among patients with FM; (2) evaluate the relationship between migraine comorbidity and SFP detected by skin biopsy; and (3) assess the independent and combined influence of migraine subtype and SFP on the multidimensional clinical profile of FM.

## 2. Methods

### 2.1. Study design and participants

This retrospective cross-sectional study was based on the analysis of a prospectively maintained clinical database of consecutive patients attending the Pain and Headache Centre, University Hospital Policlinico of Bari (Italy), between January 2018 and December 2024. Only demographic, clinical, and skin biopsy data collected during the first comprehensive outpatient evaluation were included, providing a cross-sectional assessment of the relationships among migraine comorbidity, SFP, and the multidimensional clinical profile of FM.

The study was approved by the local ethics committee (approval no. 1076/CE) and was conducted in accordance with the Declaration of Helsinki. All participants provided written informed consent for clinical assessment, skin biopsy, and anonymous use of their data for research purposes.

### 2.2. Clinical assessment

Fibromyalgia was diagnosed according to the 2016 American College of Rheumatology criteria.^25^ Clinical severity was assessed using standardized validated instruments assessing complementary dimensions of disease burden: Fibromyalgia Impact Questionnaire (FIQ); Widespread Pain Index (WPI); Symptom Severity Scale (SS); Numerical Rating Scale (NRS) for current pain intensity; Multidimensional Assessment of Fatigue (MAF); Zung Self-rating Anxiety Scale (SAS); Zung Self-rating Depression Scale (SDS); and Brief Pain Inventory (BPI), including Pain Severity and Pain Interference subscales.^1,2,5,26–28^

Headache diagnoses were established according to the International Classification of Headache Disorders, third edition (ICHD-3).^15^ Migraine was classified into 4 categories: no migraine, low-frequency episodic migraine (EM, <8 monthly migraine days), high-frequency episodic migraine (HF, 8–14 monthly migraine days), and chronic migraine (CM, ≥15 headache days/month, including ≥8 migraine days for more than 3 months). Although high-frequency episodic migraine is not formally recognized within ICHD-3, it was analysed separately because it represents a clinically relevant stage associated with increased disability and risk of progression to chronic migraine.^3^

### 2.3. Skin biopsy

All patients underwent 3-mm punch skin biopsy at the distal leg and proximal thigh according to international recommendations.^11^ The skin biopsy was performed with a 3-mm disposable punch on the thigh, 20 cm below the anterior superior iliac spine and on the distal leg (10 cm above the lateral malleolus in the area of the sural nerve), after intradermal injection of 1% xylocaine. Samples were fixed (2% paraformaldehyde lysine sodium periodate, 4°C overnight), cryopreserved, serially sectioned with a cryostat into 50-μm-thick sections and free flow immunostained with polyclonal antiprotein gene product 9.5 (Ultraclone Ltd, Rossiters Farmhouse, Wellow, Isle of Wight, UK) according to a standardised protocol. The methodology was described in detail in Devigili et al.^11^ and Lauria et al.^18^ Intraepidermal nerve fibre density was calculated on 3 nonconsecutive central sections by brightfield microscopy using a stereology workstation (Olympus BX50, PlanApo oil-objective 40x/NA = 1.0) and compared with sex- and age-adjusted normal values.^18^ Intraepidermal nerve fibre density was quantified by investigators blinded to clinical information at the Neuropathology Laboratory of the Carlo Besta Neurological Institute (Milan). Patients were classified according to the presence or absence of small fibre pathology (normal skin biopsy vs neuropathy).^18^ The distinction between isolated proximal denervation and combined proximal/distal denervation was retained only for exploratory analyses because of the limited number of patients showing length-dependent involvement.

### 2.4. Statistical analysis

Continuous variables are presented as mean ± standard deviation (SD), whereas categorical variables are expressed as frequencies and percentages. Baseline demographic characteristics were compared using one-way analysis of variance (ANOVA) for continuous variables and χ^2^ tests for categorical variables.

To facilitate clinical interpretation and reduce the number of outcome measures, the individual clinical variables were grouped a priori into 4 clinically meaningful domains: pain: WPI, NRS, and BPI Pain Severity (BPI-Severity); disability: FIQ and BPI Pain Interference (BPI-Interference); associated symptoms: SS and MAF; and psychological: Zung SAS and Zung SDS. For each domain, standardized z-scores were calculated for each component variable and averaged to obtain a composite domain score. These composite scores were used only for multivariate analyses and graphical presentation, whereas analyses of individual variables were performed separately.

The primary inferential analysis consisted of a 2-factor multivariate analysis of covariance (MANCOVA). Fixed factors were migraine subtype (no migraine, low-frequency episodic migraine, high-frequency episodic migraine, and chronic migraine); small fibre pathology (normal skin biopsy vs reduced intraepidermal nerve fibre density). Age and sex were entered as covariates because both are known to influence intraepidermal nerve fibre density and may affect the clinical severity of FM.

The dependent variables entered simultaneously into the multivariate model were the 9 individual clinical variables (FIQ, WPI, SS, NRS, MAF, SAS, SDS, BPI Pain Severity, and BPI Pain Interference). To facilitate clinical interpretation, these variables were subsequently grouped into 4 predefined domains (pain, disability, associated symptoms, and psychological) for descriptive presentation, graphical visualization, and exploratory analyses. The migraine subtype × small fibre pathology interaction was explicitly tested. Pillai's trace was selected a priori as the primary multivariate statistic because of its greater robustness to unequal group sizes and possible violations of multivariate assumptions. Wilks' lambda, Hotelling–Lawley trace, and Roy's largest root were calculated as complementary descriptive statistics.

In addition to the primary multivariate analysis, exploratory analyses were performed to better characterise the contribution of migraine subtype and SFP to individual clinical outcomes. Age- and sex-adjusted ANCOVAs were applied to the 4 clinical domains and subsequently to each individual clinical variable. Estimated marginal means with 95% confidence intervals were calculated, and *P* values were adjusted using the Holm procedure for multiple comparisons. Subsequently, the same ANCOVA model was applied separately to each individual clinical variable (FIQ, WPI, SS, NRS, MAF, SAS, SDS, BPI Pain Severity, and BPI Pain Interference).

To avoid the loss of information associated with dichotomization of skin biopsy results, additional exploratory analyses were performed using continuous IENFD measurements. Proximal and distal IENFD values were analysed separately. The relationship between continuous IENFD values and both the 4 composite clinical domains and the individual clinical variables was assessed using multivariable linear regression models. Each model included continuous IENFD (proximal or distal) as the predictor of interest; age; sex; and migraine subtype (entered as a categorical covariate). Regression coefficients (β), standard errors, 95% confidence intervals, and *P* values were reported.

These analyses were considered exploratory and were intended to determine whether the severity of small fibre involvement, rather than its dichotomous classification, was independently associated with clinical severity after accounting for demographic characteristics and migraine subtype.

Multivariate analyses were performed using complete-case analysis (listwise deletion). Regression analyses were based on all observations with complete data for the variables included in each model. Two-sided *P* values < 0.05 were considered statistically significant. For exploratory analyses involving multiple comparisons, Holm-adjusted *P* values were reported alongside unadjusted values.

All statistical analyses were performed using JMP Pro (version 2025, SAS Institute, Cary, NC) and Jamovi (2025).

## 3. Results

### 3.1. Study population

Between January 2018 and December 2024, 580 consecutive patients with FM were screened. After application of the predefined exclusion criteria (110 patients were excluded as they were undergoing current therapies with central nervous system–acting drugs or opioids at their first access; 80 patients were not considered due to different concurrent diseases not admitted for the present evaluation; 65 records were not eligible as they were not accurate or complete in several parts; and finally, 29 patients did not provide consent for the skin biopsy), 296 patients were included in the final analysis. One hundred sixty-five patients reported recurrent headache; migraine largely predominated over tension-type headache (138 vs 27 patients; χ^2^ = 78.35, *P* < 0.001). Migraine was diagnosed in 138 patients (46.6%), including 69 with low-frequency episodic migraine, 28 with high-frequency episodic migraine, and 41 with chronic migraine (Fig. 1, Table 1).

**Figure 1.**
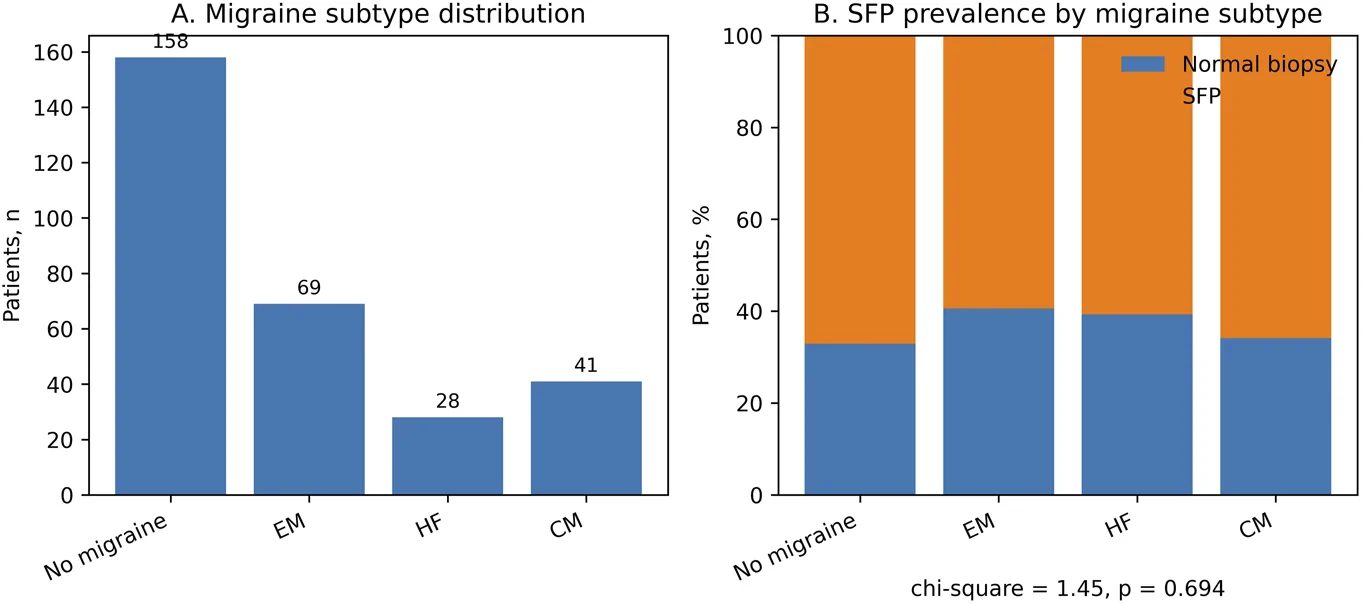
Study population and prevalence of migraine and small fibre pathology in fibromyalgia. (A) Distribution of headache diagnoses among the 296 patients with fibromyalgia, showing the prevalence of low-frequency episodic migraine (EM), high-frequency episodic migraine (HF), chronic migraine (CM), tension-type headache (TTH), and patients without headache. (B) Distribution of small fibre pathology (SFP) in patients with and without migraine. SFP prevalence did not differ significantly across migraine categories.

**Table 1:** Baseline characteristics according to migraine subtype.

Group	n	Age, y	Female	Male	SFP	Normal biopsy	*P*
No migraine	158	50.18 ± 13.67	125 (79.1)	33 (20.9)	106 (67.1)	52 (32.9)	
Low-frequency episodic migraine	69	47.32 ± 11.42	61 (88.4)	8 (11.6)	41 (59.4)	28 (40.6)	
High-frequency episodic migraine	28	46.36 ± 10.67	26 (92.9)	2 (7.1)	17 (60.7)	11 (39.3)	
Chronic migraine	41	45.83 ± 9.57	41 (100.0)	0 (0.0)	27 (65.9)	14 (34.1)	
Age ANOVA							0.103
Sex χ^2^							0.003
SFP χ^2^							0.694

Values are mean±SD or n (%).

SFP, small fibre pathology.

Reduced intraepidermal nerve fibre density was identified in 191 patients (64.5%). Only 27 patients had reduced IENFD at both proximal and distal sites; the others presented proximal IENFD reduction (Table 1).

### 3.2. Multivariate analysis

To evaluate the overall multidimensional clinical profile, a 2-factor MANCOVA was performed including migraine subtype, SFP, their interaction, age, and sex. Using Pillai's trace as the primary multivariate statistic, no significant multivariate effect was observed for migraine subtype. Age showed a significant multivariate effect, whereas sex did not (Table 2).

**Table 2:** Primary multivariate analysis (multivariate analysis of covariance).

Effect	Pillai's trace	F	df	*P*
Migraine subtype	0.069	1.43	12,723	0.148
Small fibre pathology	0.035	2.16	4239	0.075
Migraine subtype × SFP	0.047	0.96	12,723	0.490
Age	0.052	3.29	4239	0.012
Sex	0.036	2.24	4239	0.065

Primary MANCOVA including the 9 clinical variables (FIQ, WPI, SS, NRS, MAF, SAS, SDS, BPI Pain Severity, and BPI Pain Interference) as dependent variables; migraine subtype (low-frequency episodic, high-frequency episodic, and chronic migraine) and small fibre pathology as fixed factors; and age and sex as covariates. Pillai's trace was selected as the primary multivariate statistic.

BPI, Brief Pain Inventory; FIQ, Fibromyalgia Impact Questionnaire; MAF, Multidimensional Assessment of Fatigue; MANCOVA, multivariate analysis of covariance; NRS, Numerical Rating Scale; SAS, Self-rating Anxiety Scale; SDS, Self-rating Depression Scale; SFP, small fibre pathology; SS, Symptom Severity Scale; WPI, Widespread Pain Index.

These findings indicate that neither migraine subtype nor SFP status independently explained the overall multidimensional clinical profile when the 4 composite clinical domains were analysed simultaneously.

### 3.3. Exploratory analyses

Because the global multivariate analysis was not significant, subsequent analyses were considered exploratory.

### 3.4. Relationship between migraine and small fibre pathology

Baseline demographic characteristics are summarized in Table 1. Age did not differ significantly among migraine subtypes (ANOVA, *P* = 0.103), whereas all patients with chronic migraine were women (χ^2^, *P* = 0.003). The prevalence of SFP was similar across migraine categories (χ^2^ = 1.45, *P* = 0.694), indicating that peripheral small fibre pathology was not associated with migraine occurrence or chronification (Fig. 1).

This finding was confirmed by logistic regression analysis adjusted for age and sex (likelihood ratio *P* = 0.781). By contrast, increasing age independently predicted SFP (OR 1.034/year; *P* = 0.001).

### 3.5. Clinical burden associated with migraine

Patients with migraine showed significantly greater impairment than those without migraine across all predefined clinical domains (Table 3).

**Table 3:** Clinical domains according to migraine status.

Domain	N	No migraine	Migraine	F	*P*	Holm *P*
Pain	270	−0.17 ± 0.86	0.16 ± 0.69	11.09	<0.001	0.004
Disability	259	−0.22 ± 0.95	0.10 ± 0.90	5.87	0.016	0.047
Symptoms	270	−0.13 ± 0.84	0.11 ± 0.75	5.93	0.016	0.047
Psychological	258	−0.13 ± 0.92	0.13 ± 0.92	4.07	0.045	0.047

Domain scores are standardized z-score composites; *P* values are age- and sex-adjusted.

When migraine was analysed according to attack frequency (Table 4), pain scores were increased across all migraine subtypes, whereas disability, associated symptoms, and psychological burden progressively increased with migraine frequency. In particular, disability, associated symptoms, and psychopathological symptoms scores were higher in CM in respect to other migraine subtypes (Fig. 2, Table 4).

**Table 4:** Clinical domains according to migraine subtype.

Domain	N	No migraine	Low-frequency episodic migraine	High-frequency episodic migraine	Chronic migraine	F	*P*	Holm *P*
Pain	270	−0.17 ± 0.86	0.13 ± 0.62	0.20 ± 0.78	0.16 ± 0.77	3.73	0.012	0.047
Disability	259	−0.22 ± 0.95	−0.07 ± 0.90	0.07 ± 0.97	0.39 ± 0.80	3.67	0.013	0.047
Symptoms	270	−0.13 ± 0.84	0.04 ± 0.70	−0.04 ± 0.75	0.33 ± 0.79	3.49	0.016	0.047
Psychological	258	−0.13 ± 0.92	0.03 ± 0.92	−0.07 ± 0.94	0.41 ± 0.86	3.13	0.026	0.047

Domain scores are standardized z-score composites; *P* values are age- and sex-adjusted.

**Figure 2.**
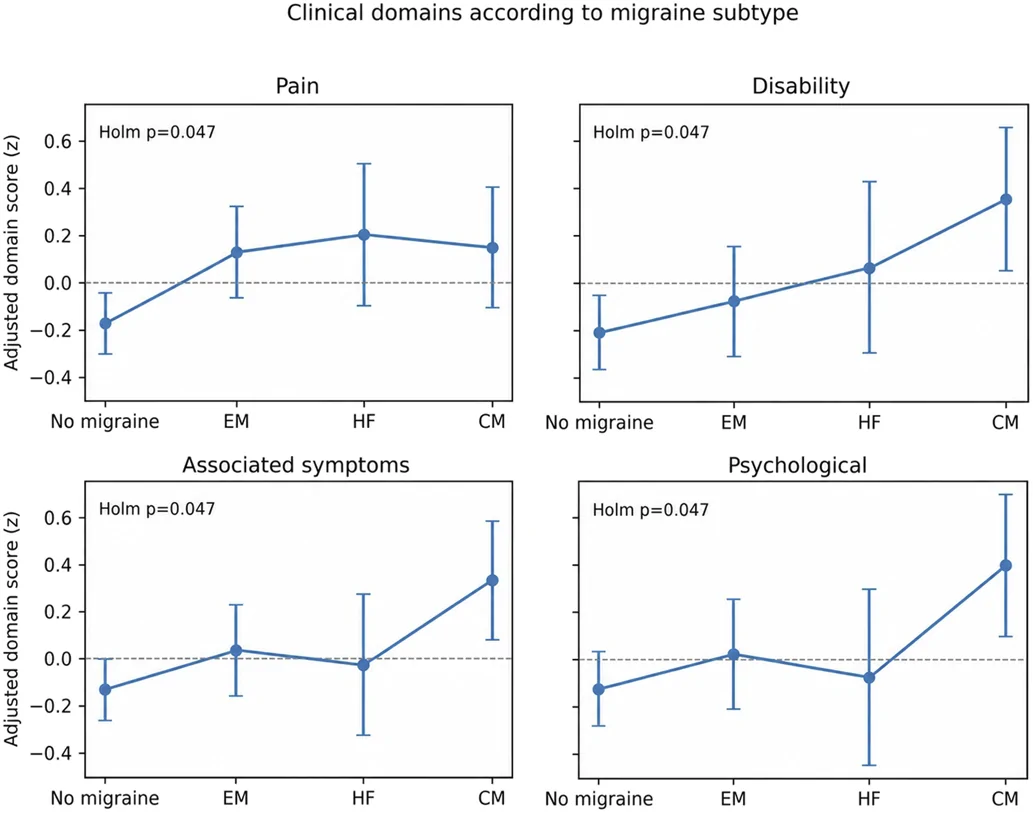
Clinical domains according to migraine subtype. Mean age-adjusted scores for the predefined clinical domains according to migraine subtype (no migraine, EM: low-frequency episodic migraine, HF: high-frequency episodic migraine, and CM: chronic migraine). Chronic migraine was associated with the highest disability, associated symptom burden, and psychological impairment.

### 3.6. Clinical burden associated with small fibre pathology

When analysed independently, SFP was not associated with significant differences in the predefined clinical domains after adjustment for age and sex (Fig. 3, Table 5). Thus, peripheral small fibre pathology alone did not identify a distinct multidimensional clinical phenotype.

**Figure 3.**
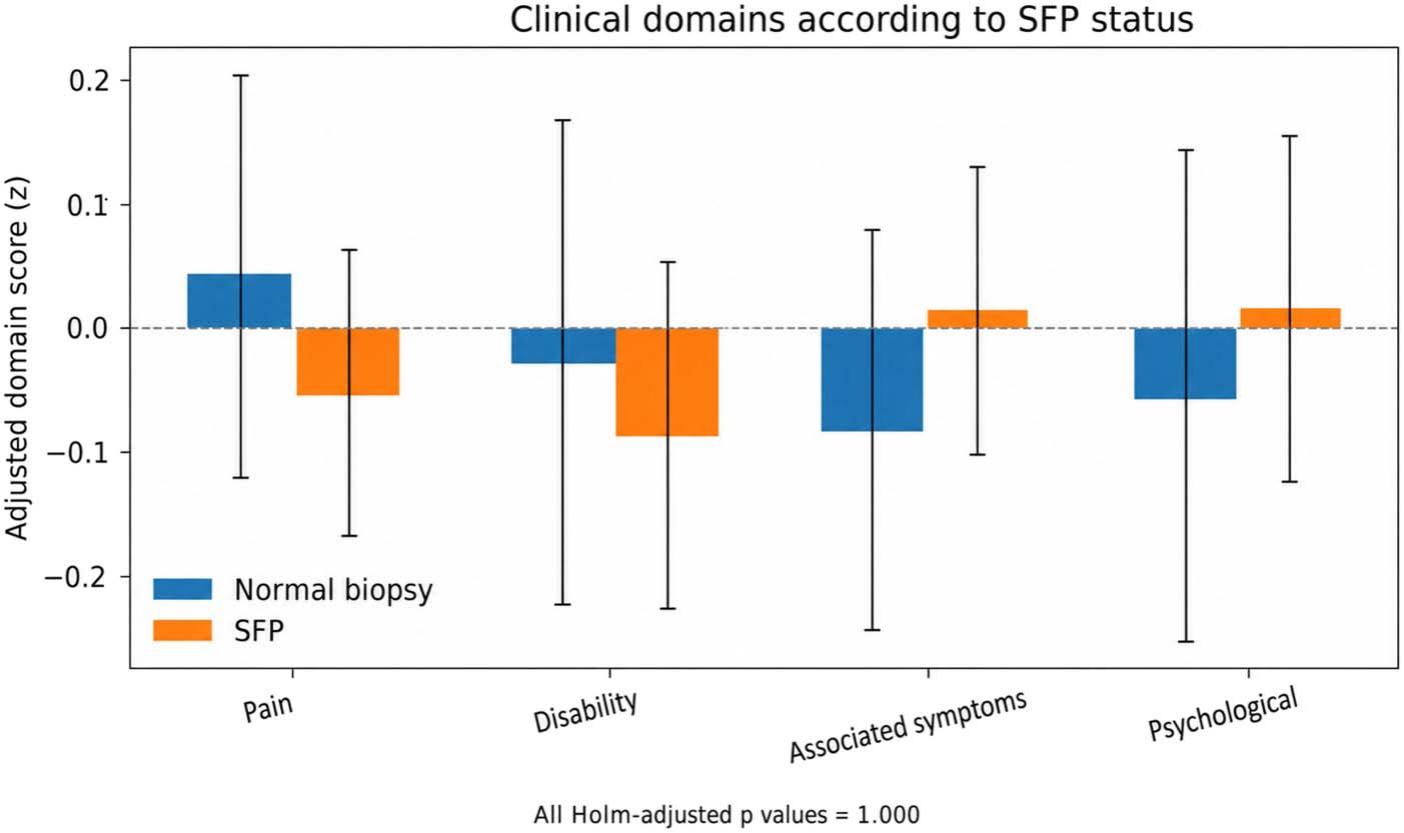
Clinical domains according to small fibre pathology. Comparison of age-adjusted clinical domain scores between patients with normal skin biopsy and patients with small fibre pathology. No significant differences were observed across the predefined domains after adjustment for age and sex.

**Table 5:** Clinical domains according to small fibre pathology status.

Domain	N	Normal biopsy	SFP	F	*P*	Holm *P*
Pain	270	−0.01 ± 0.73	−0.03 ± 0.84	0.88	0.348	1.000
Disability	259	−0.07 ± 0.79	−0.07 ± 1.01	0.23	0.632	1.000
Symptoms	270	−0.14 ± 0.80	0.04 ± 0.80	0.91	0.341	1.000
Psychological	258	−0.07 ± 0.93	0.03 ± 0.93	0.34	0.561	1.000

Domain scores are standardized z-score composites; *P* values are age- and sex-adjusted.

SFP, small fibre pathology.

### 3.7. Exploratory subgroup analysis

Considering the increasing burden of FM in patients with chronic migraine, we performed an additional exploratory comparison in patients with concomitant CM and SFP and the remainder of the cohort. After adjustment for age and sex, this subgroup showed higher adjusted disability and associated symptoms scores, whereas differences in pain and psychological domains did not remain consistently significant after correction for multiple comparisons (Table 6; Fig. 4). Because the global migraine subtype × SFP interaction was not significant in the primary multivariate analysis, these subgroup findings should be considered hypothesis-generating and interpreted with caution.

**Table 6:** Chronic migraine + small fibre pathology subgroup analysis.

Domain	N	Others adjusted mean (95% CI)	CM + SFP adjusted mean (95% CI)	F	*P*	Holm *P*
Pain	270	−0.04 (−0.14, 0.06)	0.17 (−0.14, 0.48)	1.61	0.206	0.206
Disability	259	−0.12 (−0.24, −0.00)	0.44 (0.08, 0.80)	8.39	0.004	0.016
Associated symptoms	270	−0.06 (−0.15, 0.04)	0.36 (0.05, 0.66)	6.41	0.012	0.036
Psychological domain	258	−0.05 (−0.17, 0.07)	0.36 (0.00, 0.72)	4.48	0.035	0.070

Adjusted means and 95% CI; *P* values are age- and sex-adjusted.

CM, chronic migraine; SFP, small fibre pathology.

**Figure 4.**
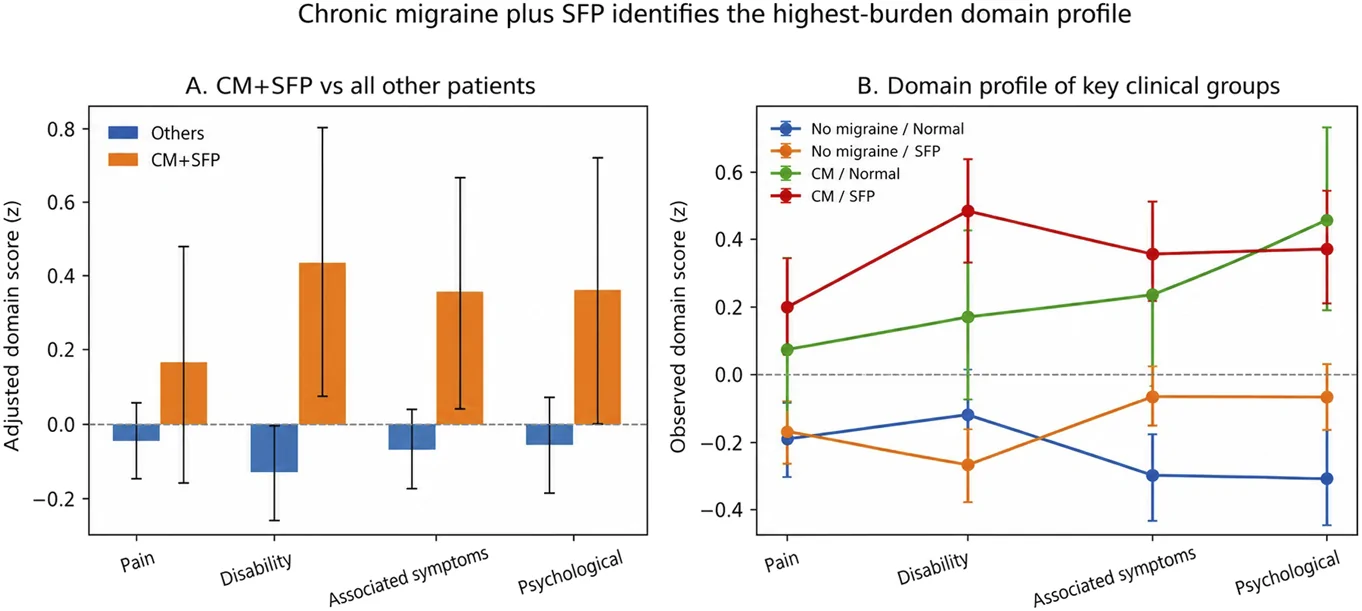
Clinical profile of patients with chronic migraine (CM) and small fibre pathology. (A) Age-adjusted domain scores comparing patients with chronic migraine and concomitant small fibre pathology with the remaining clinical subgroups. The coexistence of chronic migraine and SFP identified the subgroup with the greatest disability and associated symptom burden. (B) Canonical discriminant analysis illustrating the multidimensional clinical distribution of the 8 groups defined by migraine subtype and small fibre pathology. Separation among groups reflects the combined contribution of multiple correlated clinical variables rather than isolated differences in single outcome measures. SFP, small fibre pathology.

### 3.8. Exploratory analysis of continuous intraepidermal nerve fibre density

Continuous proximal and distal IENFD values were additionally analysed using multivariable linear regression models adjusted for age, sex, and migraine subtype. No significant independent associations were observed between continuous IENFD values and any of the individual clinical variables or composite clinical domains. These findings indicate that the severity of intraepidermal nerve fibre loss was not independently associated with clinical severity after accounting for demographic characteristics and migraine subtype. Detailed regression results are reported in Supplementary Table S8, https://links.lww.com/PR9/A454.

## 4. Discussion

The principal finding of this study is that migraine, particularly its chronic form, is associated with greater clinical burden in patients with FM. By contrast, neither small fibre pathology nor the interaction between migraine subtype and small fibre pathology demonstrated a significant effect on the overall multidimensional clinical profile in the primary multivariate analysis. Exploratory analyses suggested that patients with concomitant chronic migraine and small fibre pathology may represent a subgroup with greater disability and associated symptom burden; however, these observations should be considered hypothesis-generating.

### 4.1. Migraine in the fibromyalgia cohort

Approximately half of the patients with FM in our cohort had migraine, and half of these suffered from high-frequency episodic or chronic forms, confirming that migraine is one of the most frequent neurological comorbidities of FM.^4,7,9,19,20^ Most previous studies have focused on the impact of FM on headache, consistently showing that FM comorbidity is associated with increased migraine frequency, treatment resistance, and greater disability.^7^ Conversely, fewer studies have investigated how migraine influences the clinical expression of FM. Our findings indicate that the burden is not simply related to the presence of migraine but is mainly driven by its chronic form. Although pain dimension scores were higher in all migraine subtypes, patients with chronic migraine showed greater disability, more severe associated symptoms, and psychopathological aspects, suggesting that persistent migraine activity contributes to the multidimensional burden of FM.

This interpretation is consistent with the concept of chronic migraine as a disorder characterised by sustained central sensitization and increased vulnerability to pain amplification.^6^ Repeated activation of central nociceptive networks may contribute to greater interference with daily activities and psychological distress, even in patients already affected by widespread pain. Although psychopathological symptoms are highly prevalent in both FM and migraine,^17,24^ our results suggest that the coexistence of chronic migraine may further amplify this dimension of disease burden. Because patients with major psychiatric disorders were excluded from the study, the observed high scores within psychological dimension were unlikely to be explained solely by preexisting psychiatric comorbidity.

Interestingly, tension-type headache was uncommon in our cohort despite its recognized association with FM in headache-based populations.^7^ This difference probably reflects the distinct referral pathways of tertiary headache centres compared with specialized pain clinics. Overall, these findings indicate that chronic migraine, rather than migraine per se, represents the clinical condition most strongly associated with a more severe FM phenotype, supporting careful identification of migraine frequency in patients referred for FM evaluation.

### 4.2. Small fibre pathology in the fibromyalgia cohort

Reduced intraepidermal nerve fibre density was observed in nearly two-thirds of our patients, confirming previous reports that small fibre pathology is a frequent finding in FM.^8,21,23^ In confirmation of previous reports, patients with FM were affected by non–length-dependent neuropathy, with a minority of them with additional distal site involvement without the presence of distal–proximal gradient of typical small fibre neuropathy.^8,11,23^ Nevertheless, neither the dichotomous classification of SFP nor continuous IENFD measurements were independently associated with the multidimensional clinical profile after adjustment for age, sex, and migraine subtype. These findings support the concept that reduced IENFD alone does not define a clinically distinct phenotype, despite representing an important biological feature in a substantial proportion of patients with FM.

The absence of independent associations between continuous IENFD values and clinical severity further suggests that the extent of small fibre loss is not linearly related to symptom burden. This observation agrees with previous studies reporting a dissociation between structural small fibre abnormalities and the clinical expression of FM, supporting the hypothesis that peripheral nerve pathology represents only one component of a complex multidimensional disorder.^8,21^

### 4.3. Multidimensional integration between small fibre pathology and migraine

The prevalence of SFP was similar across migraine subtypes, indicating that peripheral nerve pathology is not specifically associated with migraine occurrence or chronification. Exploratory subgroup analyses identified patients with concomitant chronic migraine and small fibre pathology as showing the highest adjusted disability and associated symptom scores. However, because the primary MANCOVA did not demonstrate a significant migraine subtype × SFP interaction, these observations should be interpreted cautiously. Rather than demonstrating a distinct biological phenotype, they identify a subgroup deserving further investigation in larger prospective cohorts. In fact, chronic migraine may represent the dominant clinical marker of central sensitization,^6^ whereas SFP may amplify the expression of FM only in this subgroup of patients. Thus, the coexistence of chronic migraine and SFP may identify a subgroup of patients characterised by greater functional impairment and symptom burden, potentially reflecting the interaction between persistent central sensitization and ongoing peripheral nociceptive input. Recent evidence of the pivotal role of trigeminal afferents activation by CGRP in migraine pathogenesis support the relevance of peripheral–central circuit interactions in the attack onset and persistence.^16^ Future studies integrating structural, neurophysiological, and longitudinal biomarkers will be necessary to establish whether the coexistence of persistent migraine activity and peripheral small fibre abnormalities has prognostic or therapeutic implications.

### 4.4. Clinical implications

From a clinical perspective, the present findings reinforce the importance of systematically assessing migraine in patients with FM. Chronic migraine was consistently associated with greater disability and symptom burden across exploratory analyses, whereas skin biopsy abnormalities alone did not identify patients with a distinct clinical presentation. Whether patients presenting both chronic migraine and small fibre pathology represent a subgroup requiring tailored therapeutic strategies remains to be established.

Treatment of migraine may also reduce the global burden of FM, as shown in recent studies evaluating the effects of anti-CGRP drugs and botulinum toxin.^9,22^

### 4.5. Limitations and future perspectives

This study has several limitations. First, its retrospective cross-sectional design does not allow conclusions regarding causal relationships between chronic migraine, small fibre pathology, and the evolution of FM. Whether peripheral small fibre abnormalities precede, follow, or develop in parallel with central sensitization cannot be established from the present data.

Second, skin biopsy was performed only at the initial evaluation, preventing assessment of longitudinal relationships between changes in intraepidermal nerve fibre density and clinical evolution. Likewise, migraine frequency was classified at baseline and may have changed over time.

Third, although the overall cohort was relatively large, the subdivision into migraine subtypes resulted in relatively small numbers within some groups, particularly the high-frequency migraine subgroup. Therefore, the subgroup analyses should be interpreted cautiously and require confirmation in larger prospective cohorts.

Although exploratory analyses suggested that patients with concomitant chronic migraine and small fibre pathology may experience greater disability, the primary multivariate analysis did not demonstrate a significant interaction between these factors. Therefore, these observations should be considered exploratory and require confirmation in larger prospective studies.

## 5. Conclusions

Our findings indicate that migraine, particularly its chronic form, is associated with greater clinical burden in FM. By contrast, small fibre pathology, whether analysed as a dichotomous variable or as continuous intraepidermal nerve fibre density, was not independently associated with the multidimensional clinical profile.

Although exploratory analyses suggested that patients with concomitant chronic migraine and small fibre pathology may experience greater disability and associated symptom burden, these observations were not supported by the primary multivariate analysis and should therefore be considered hypothesis-generating.

Overall, our results support the concept that migraine represents a clinically relevant determinant of disease burden in FM, whereas the contribution of peripheral small fibre abnormalities to clinical heterogeneity remains uncertain. Prospective longitudinal studies integrating clinical, neurophysiological, and structural biomarkers will be necessary to clarify the role of peripheral nerve pathology in the evolution and treatment of FM.

## Disclosures

The authors have no conflict of interest to declare.

## Supplemental digital content

Supplemental digital content associated with this article can be found online at https://links.lww.com/PR9/A454
