# Supplementary material for: Central and peripheral dimensions of fibromyalgia: a multivariate clinical profiling of migraine and small fibre pathology
Source: Pain Rep. 2026 Oct 28;11(6):e1510. doi: 10.1097/PR9.0000000000001510 (PMC13719114; doi:10.1097/PR9.0000000000001510)
Supplement: Supplementary file 1 [file painreports-11-e1510-s001.pdf]

Supplementary Material

Central and Peripheral Dimensions of Fibromyalgia: A Multivariate Clinical Profiling Study

This supplementary file reports additional variable-level analyses performed on the complete-case dataset used for the multivariable clinical profiling analyses. The analyses are intended to document the contribution of individual clinical variables composing the predefined domains and to support interpretation of the multivariate findings.

Supplementary Methods

Follow-up analyses of covariance (ANCOVAs) were performed for each clinical variable entered into the multivariate model. The model included migraine subtype (No migraine, Episodic Migraine, High-frequency Migraine and Chronic Migraine), small fibre pathology (normal skin biopsy vs neuropathy), their interaction, age and sex. Type III sums of squares were used. Effect sizes are reported as partial eta squared.

Canonical discriminant analysis was performed on age- and sex-adjusted standardized residuals of the nine individual clinical variables. This exploratory analysis was used to visualize the multivariate separation of the eight groups defined by migraine subtype and small fibre pathology.

Supplementary Results

The complete-case dataset included 199 patients. The dependent variables were FIQ, WPI, SS, NRS, MAF, SAS, SDS, BPI Pain Severity and BPI Pain Interference.

Supplementary Table S1. Validation of model assumptions.

Assumption	Method	Result
Complete-case sample	Listwise deletion	n = 199
Homogeneity of covariance matrices	Box M test, approximate chi-square	chi-square = 554.26, df = 315, p = <0.001
Equality of variances	Levene tests across 8 groups	See Table S3; isolated deviations interpreted cautiously
Multicollinearity	Pairwise correlation matrix	No pairwise correlation exceeded 0.90; see Table S6
Independence	Study design	Each participant contributed one baseline observation

Supplementary Table S2. Follow-up ANCOVAs for individual clinical variables.

Values are Type III F statistics with p values and partial eta squared. All models include age and sex.

Variable	Effect	df	F	p	Partial eta2	Interpretation
FIQ	Migraine subtype	3	0.56	0.645	0.009	
FIQ	SFP	1	0.09	0.760	0.000	
FIQ	Migraine x SFP	3	0.43	0.733	0.007	
FIQ	Age	1	1.92	0.168	0.010	
FIQ	Sex	1	0.19	0.660	0.001	
WPI	Migraine subtype	3	1.32	0.268	0.021	
WPI	SFP	1	0.51	0.474	0.003	
WPI	Migraine x SFP	3	0.57	0.634	0.009	
WPI	Age	1	3.46	0.064	0.018	
WPI	Sex	1	0.85	0.358	0.004	
SS	Migraine subtype	3	2.20	0.089	0.034	
SS	SFP	1	4.02	0.046	0.021	nominal p<0.05
SS	Migraine x SFP	3	0.51	0.676	0.008	
SS	Age	1	5.03	0.026	0.026	nominal p<0.05
SS	Sex	1	0.47	0.493	0.002	
NRS	Migraine subtype	3	0.59	0.624	0.009	
NRS	SFP	1	0.80	0.372	0.004	

Variable	Effect	df	F	p	Partial eta2	Interpretation
NRS	Migraine x SFP	3	0.69	0.559	0.011	
NRS	Age	1	5.88	0.016	0.030	nominal p<0.05
NRS	Sex	1	1.48	0.225	0.008	
MAF	Migraine subtype	3	0.89	0.446	0.014	
MAF	SFP	1	0.13	0.714	0.001	
MAF	Migraine x SFP	3	0.90	0.445	0.014	
MAF	Age	1	0.22	0.641	0.001	
MAF	Sex	1	3.50	0.063	0.018	
SAS	Migraine subtype	3	2.60	0.053	0.040	
SAS	SFP	1	2.85	0.093	0.015	
SAS	Migraine x SFP	3	0.92	0.433	0.014	
SAS	Age	1	0.03	0.860	0.000	
SAS	Sex	1	0.09	0.766	0.000	
SDS	Migraine subtype	3	2.81	0.041	0.043	nominal p<0.05
SDS	SFP	1	8.32	0.004	0.042	nominal p<0.05
SDS	Migraine x SFP	3	1.80	0.149	0.028	
SDS	Age	1	0.31	0.576	0.002	
SDS	Sex	1	0.02	0.883	0.000	
BPI Severity	Migraine subtype	3	0.84	0.472	0.013	
BPI Severity	SFP	1	0.68	0.410	0.004	
BPI Severity	Migraine x SFP	3	0.97	0.406	0.015	
BPI Severity	Age	1	6.26	0.013	0.032	nominal p<0.05
BPI Severity	Sex	1	0.92	0.339	0.005	
BPI Interference	Migraine subtype	3	2.35	0.074	0.036	
BPI Interference	SFP	1	4.25	0.041	0.022	nominal p<0.05
BPI Interference	Migraine x SFP	3	1.29	0.279	0.020	
BPI Interference	Age	1	1.53	0.217	0.008	
BPI Interference	Sex	1	1.47	0.227	0.008	

Supplementary Table S3. Levene tests for equality of variances across migraine/SFP groups.

Variable	Levene F	p
FIQ	0.37	0.918
WPI	1.71	0.108
SS	0.66	0.707
NRS	0.86	0.540
MAF	0.90	0.506
SAS	1.11	0.357
SDS	1.03	0.415
BPI Severity	1.45	0.187
BPI Interference	0.73	0.648

Supplementary Table S4. Standardized canonical coefficients.

Variable	Function 1	Function 2
FIQ	-0.606	-0.322
WPI	-0.312	0.048
SS	0.420	-0.371
NRS	-0.073	0.414
MAF	0.319	0.244
SAS	0.151	-0.358
SDS	0.485	0.066
BPI Severity	-0.497	-0.935
BPI Interference	0.500	0.145

Supplementary Table S5. Canonical group centroids.

Migraine subtype	SFP	Function 1	Function 2
CM	neuropathy	-0.086	-0.779
CM	normal skin biopsy	0.571	-0.516
ESA	neuropathy	0.031	0.013
ESA	normal skin biopsy	0.228	0.310
HF	neuropathy	-0.355	-0.442
HF	normal skin biopsy	-0.438	0.133
N	neuropathy	0.233	0.170
N	normal skin biopsy	-0.697	0.218

Supplementary Table S6. Correlation matrix among clinical variables entered into the model.

Variable	FIQ	WPI	SS	NRS	MAF	SAS	SDS	BPI Severity	BPI Interference
FIQ	1.00	0.32	0.31	0.54	0.60	0.50	0.52	0.50	0.54
WPI	0.32	1.00	0.39	0.29	0.32	0.24	0.22	0.23	0.25
SS	0.31	0.39	1.00	0.16	0.23	0.32	0.39	0.23	0.24
NRS	0.54	0.29	0.16	1.00	0.39	0.23	0.20	0.61	0.34
MAF	0.60	0.32	0.23	0.39	1.00	0.40	0.37	0.44	0.40
SAS	0.50	0.24	0.32	0.23	0.40	1.00	0.66	0.25	0.49
SDS	0.52	0.22	0.39	0.20	0.37	0.66	1.00	0.22	0.57
BPI Severity	0.50	0.23	0.23	0.61	0.44	0.25	0.22	1.00	0.45
BPI Interference	0.54	0.25	0.24	0.34	0.40	0.49	0.57	0.45	1.00

Supplementary Table S7. Descriptive values by migraine subtype and SFP status.

Values are mean ± SD.

Migraine subtype	SFP	n	FIQ	WPI	SS	NRS	MAF	SAS	SDS	BPI Severity	BPI Interference
N	normal skin biopsy	26	60.65 ± 14.93	13.42 ± 3.91	6.08 ± 2.23	8.31 ± 1.41	29.19 ± 15.53	42.96 ± 9.17	42.04 ± 8.82	29.50 ± 5.53	15.23 ± 6.34
N	neuropathy	66	59.89 ± 15.35	14.17 ± 4.27	7.15 ± 2.32	8.12 ± 1.83	31.26 ± 12.66	46.15 ± 7.27	47.35 ± 7.03	28.79 ± 7.88	18.36 ± 6.85
ESA	normal skin biopsy	25	57.12 ± 13.24	14.32 ± 4.30	6.88 ± 1.69	7.84 ± 1.62	30.88 ± 12.64	45.28 ± 8.01	45.52 ± 7.17	27.52 ± 5.53	18.76 ± 5.15
ESA	neuropathy	30	59.87 ± 17.73	15.00 ± 3.71	7.63 ± 1.79	8.30 ± 1.70	30.10 ± 15.07	45.17 ± 9.41	45.90 ± 8.26	29.67 ± 7.85	18.03 ± 6.27
HF	normal skin biopsy	9	56.78 ± 15.16	15.78 ± 3.63	7.11 ± 1.83	8.67 ± 1.41	24.78 ± 18.46	42.33 ± 6.98	43.22 ± 10.81	30.22 ± 6.44	18.00 ± 9.41
HF	neuropathy	13	62.54 ± 10.85	15.69 ± 2.50	6.85 ± 2.27	8.38 ± 1.89	35.77 ± 10.65	46.46 ± 6.15	46.23 ± 7.88	34.38 ± 9.06	19.62 ± 7.91
CM	normal skin biopsy	11	63.36 ± 16.65	12.55 ± 5.54	7.73 ± 2.76	8.09 ± 2.07	35.91 ± 13.28	50.73 ± 7.21	49.73 ± 5.68	31.55 ± 5.73	20.09 ± 6.47
CM	neuropathy	19	68.05 ± 14.91	15.11 ± 2.81	8.16 ± 2.09	8.63 ± 1.07	34.05 ± 12.46	49.21 ± 7.63	48.26 ± 7.45	33.68 ± 5.20	19.84 ± 5.39

Supplementary Table S8. Association between continuous intraepidermal nerve fibre density (IENFD) and clinical variables

Clinical variable	Proximal IENFD β	p	Distal IENFD β	p
FIQ	NS	>0.05	NS	>0.05
WPI	NS	>0.05	NS	>0.05
Symptom Severity (SS)	NS	>0.05	NS	>0.05
NRS pain	NS	>0.05	NS	>0.05
MAF	NS	>0.05	NS	>0.05
SAS	NS	>0.05	NS	>0.05
SDS	NS	>0.05	NS	>0.05
BPI Pain Severity	NS	>0.05	NS	>0.05
BPI Pain Interference	NS	>0.05	NS	>0.05

Multivariable linear regression models were adjusted for age, sex and migraine subtype. Proximal and distal intraepidermal nerve fibre density (IENFD) were analysed separately. No significant independent associations were observed between continuous IENFD values and any clinical variable.

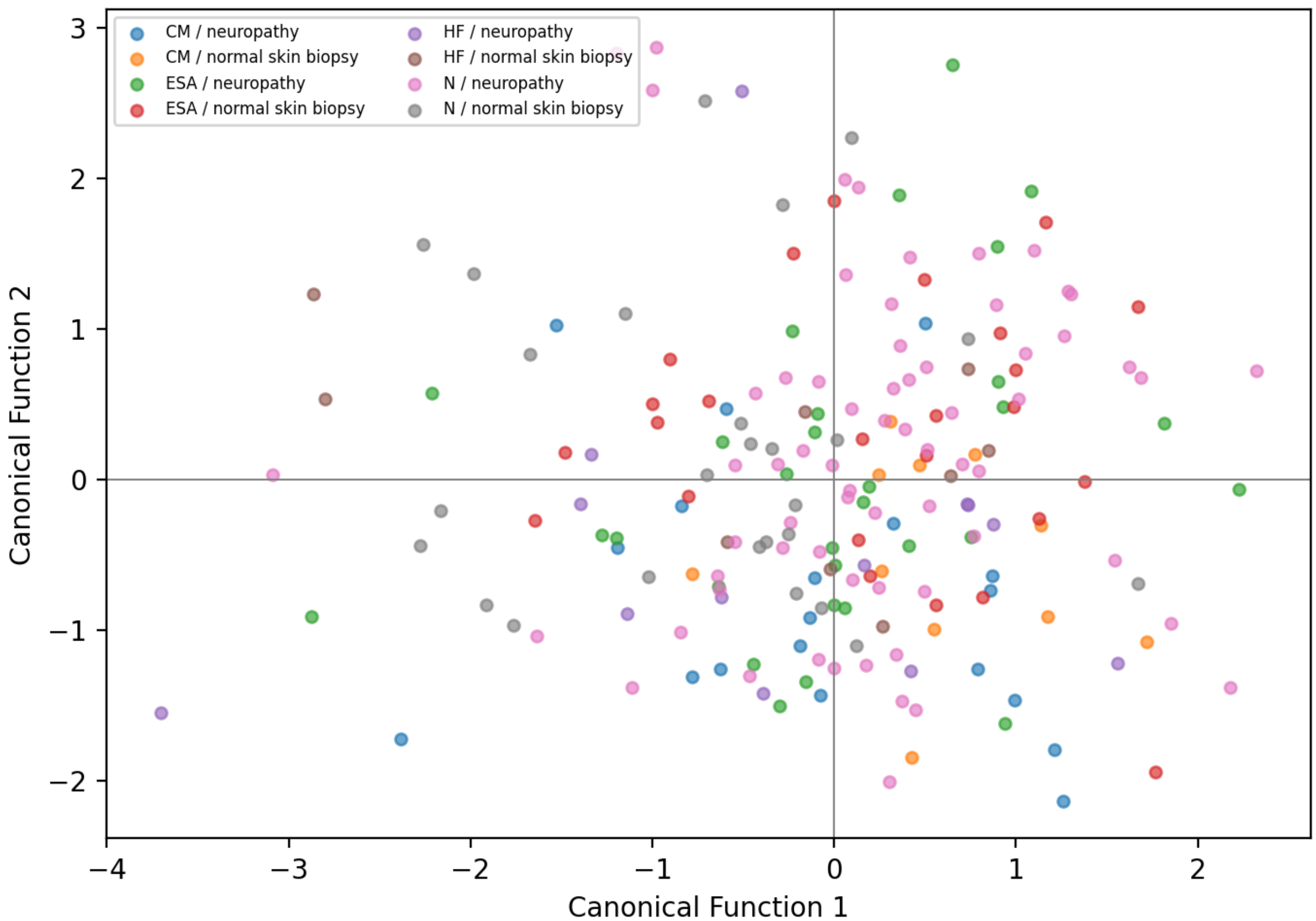

Supplementary Figure S1. Canonical discriminant analysis based on age- and sex-adjusted standardized residuals of the nine clinical variables.

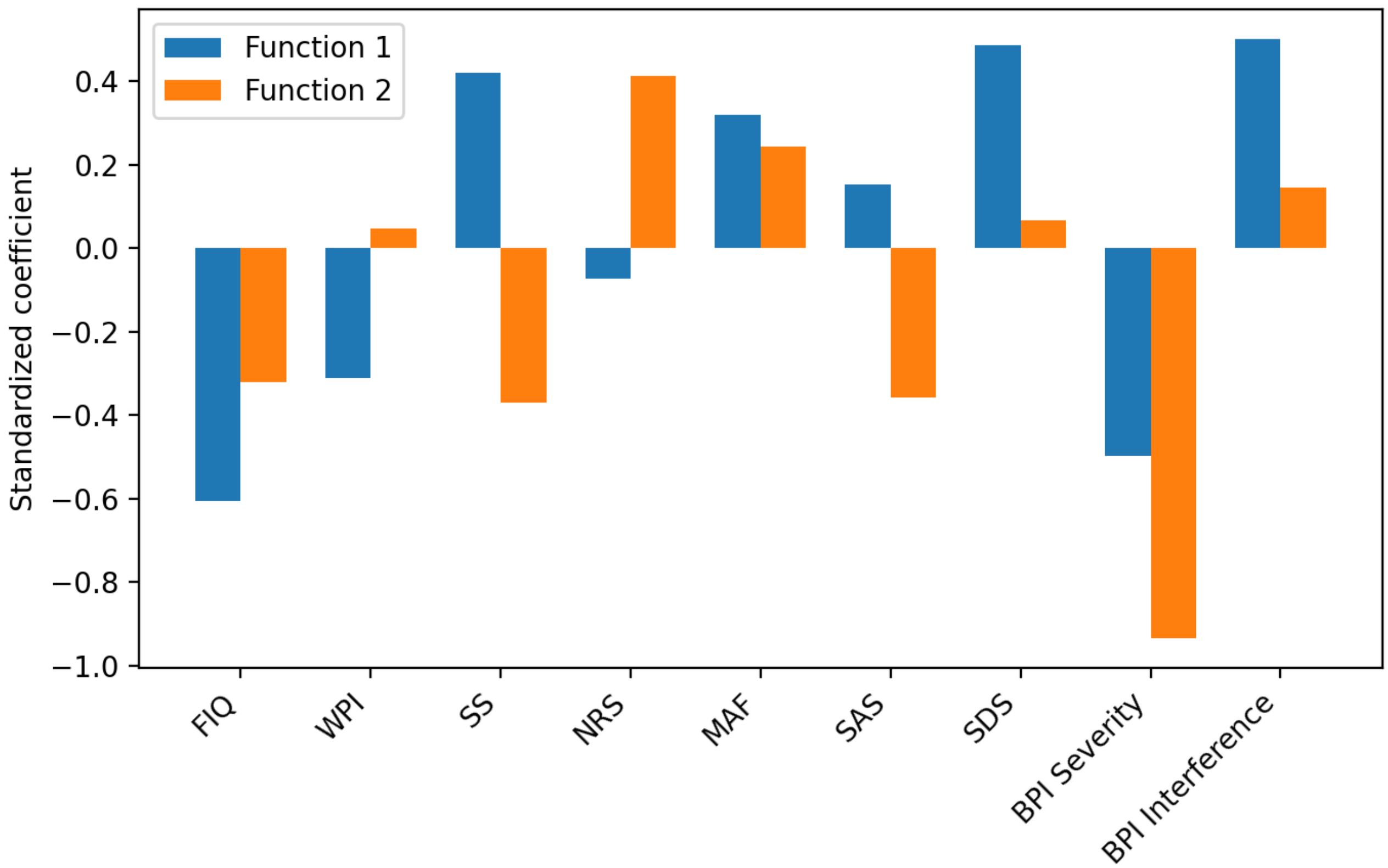

Supplementary Figure S2. Standardized canonical coefficients for Functions 1 and 2.

Supplementary interpretation

The variable-level analyses show that the clinical burden associated with migraine subtype and SFP is distributed across multiple partially correlated outcomes. Therefore, these results should be interpreted as exploratory follow-up analyses supporting the multidimensional clinical profile rather than as independent confirmatory endpoints.

STROBE Checklist

Titolo

STROBE Statement—Checklist of items that should be included in reports of cross-sectional studies

Manuscript:

Central and Peripheral Dimensions of Fibromyalgia: A Multivariate Clinical Profiling Study of Migraine and Small Fibre Pathology Comorbidity

---

La tabella

STROBE item	Recommendation	Page
Title and abstract	Study design indicated	1–3
Background/rationale	Scientific background explained	4–5
Objectives	Objectives and hypotheses	5
Study design	Cross-sectional design described	5–6
Setting	Pain and Headache Centre, 2018–2024	5
Participants	Inclusion/exclusion criteria	9–10
Variables	Outcomes and predictors defined	6–9
Data sources	Clinical scales and skin biopsy	6–8
Bias	Consecutive cohort and standardized assessments	5–10
Study size	Final sample (296) described	9–10
Quantitative variables	Definition of migraine categories and IENFD	6–8
Statistical methods	MANCOVA, ANCOVA, regression analyses	7–9
Participants	Flow diagram	Figure 1
Descriptive data	Table 1	10
Outcome data	Tables 2–6	10–12
Main results	Multivariate and exploratory analyses	10–12
Other analyses	Continuous IENFD regression; Supplementary Material	11–12
Key results	Discussion	13
Limitations	Limitations discussed	16
Interpretation	Clinical interpretation	13–17
Generalisability	Conclusions	17
Funding	Funding statement	17

All applicable STROBE items have been addressed in the manuscript. Items not applicable to the present cross-sectional observational design were marked as not applicable where appropriate
